# EvaLuation Using Cardiac Insertable Devices And TelephonE in Hypertrophic Cardiomyopathy (ELUCIDATE HCM): A prospective observational study on incidence of arrhythmias

**DOI:** 10.1111/jce.14792

**Published:** 2020-11-04

**Authors:** Peter Magnusson, Stellan Mörner

**Affiliations:** ^1^ Centre for Research and Development Uppsala University Region Gävleborg Sweden; ^2^ Department of Medicine, Cardiology Research Unit Karolinska Institutet Stockholm Sweden; ^3^ Department of Public Health and Clinical Medicine Umeå University Umeå Sweden

**Keywords:** arrhythmia, atrial fibrillation, hypertrophic cardiomyopathy, insertable cardiac monitor, non‐sustained ventricular tachycardia

## Abstract

**Background:**

Hypertrophic cardiomyopathy (HCM) is a heterogeneous disease associated with arrhythmias. Non‐sustained ventricular tachycardia (NSVT) is a risk factor for sudden cardiac death and part of the current risk stratification. Furthermore, atrial fibrillation (AF), which increases the risk of stroke, is believed to be common in HCM patients. Routine ambulatory monitoring captures the rhythm only periodically over 24–48 h; thus, the true burden of arrhythmia is unknown. The insertable cardiac monitor (ICM) should help determine a more realistic arrhythmia assessment in HCM patients.

**Objective:**

The purpose of this study was to ascertain the incidence of NSVT, AF, and bradycardia in unselected HCM patients by the use of an ICM.

**Methods:**

Thirty adults, mean age 49.9 ± 12.3 years, 25 (83.3%) males were implanted with a Confirm Rx ICM. The monitoring application was installed on the patient's smartphone, which allowed for patient activation in case of symptoms. The ICM was programmed as follows: ventricular tachycardia (VT) ≥ 160 beats per minute (bpm) for ≥8 intervals, AF ≥ 2 min of duration, and bradycardia ≤ 40 bpm or pause ≥ 3.0 s.

**Results:**

The mean calculated 5‐year risk was 2.3%, and 29/30 of the patients had a risk <4%. During follow‐up, AF was found in nine patients (30.0%). At least one episode of NSVT was detected in seven patients (23.3%). In 13 patients (43.3%), sinoatrial block/sinus arrest/sinus bradycardia were seen. No arrhythmia was detected in nine patients (30.0%).

**Conclusion:**

In this first prospective study using an ICM, the arrhythmia burden in HCM patients yielded 30.0% AF and 23.3% NSVT.

**Abbreviations**
AF: atrial fibrillation
HCM: hypertrophic cardiomyopathy
ICM: insertable cardiac monitor
NSVT: non‐sustained VT
VT: ventricular tachycardia

## 1 INTRODUCTION

The hypertrophic cardiomyopathy (HCM) phenotype in adults requires at least 15 mm thickness in any segment of the myocardial wall, which is not explained by other myocardial diseases or abnormal loading conditions due to hypertension or aortic stenosis.^1^ The prevalence is typically cited as 1:500, but it could be higher if genotypes are also included.^1^ Symptoms of HCM are unspecific, such as dyspnea, chest discomfort, palpitations, and dizziness. Arrhythmias, both bradycardia and tachycardia, are reported in HCM patients. HCM patients may be particularly vulnerable to symptoms associated with atrial fibrillation (AF) because of the elevated heart rate and compromised atrial filling. Furthermore, AF associated with ischemic stroke or systemic embolization is a major cause of death in HCM, which makes attention to effective arrhythmia detection methods so important.^2^ In fact, according to the European Society of Cardiology (ESC) guidelines, a history of AF, even without any CHA_2_DS_2_‐VASc risk factors, is an indication for anticoagulation.^3^

Sudden cardiac death is mainly caused by ventricular tachycardia (VT) or ventricular fibrillation, even though bradycardia may be implicated as well. An implantable cardioverter‐defibrillator (ICD) effectively restores rhythm with an annual rate of appropriate therapy of 4.8%, according to a meta‐analysis.^4^ Because ICDs are prone to complications, including inappropriate shocks, careful selection of patients for primary prevention based on risk factors is warranted. The presence of non‐sustained VT (NSVT) is considered a risk factor in both European^3^ and American^5^ guidelines based on several studies.^6^, ^7^, ^8^, ^9^, ^10^ Both higher age and increased maximum wall thickness are linked to NSVT.^9^, ^11^ NSVT may be revealed during an ambulatory electrocardiogram (ECG), telemetry in the ward, or during an exercise test. Guidelines advocate ambulatory monitoring after the onset of palpitations (for 48 h), every 6–12 months if the patient has an enlarged left atrial diameter (which predisposes the patient to AF and sudden cardiac death^6^) but is in sinus rhythm, or every 12–24 months for remaining patients.^3^ The presence of NSVT/AF is unknown during the unmonitored periods. The actual implementation of these recommendations is unknown, and monitoring may be somewhat arbitrary.

The insertable cardiac monitor (ICM) Confirm Rx (Abbott/St Jude Medical) provides long‐term monitoring of atrial and ventricular arrhythmias in addition to bradycardia.^12^ This device could potentially reveal the true incidence of arrhythmia in patients with HCM, which is the rationale of the present study: EvaLuation Using Cardiac Insertable Devices And TelephonE in Hypertrophic Cardiomyopathy (ELUCIDATE HCM).^13^ The study objectives were to evaluate the incidence of NSVT and AF over 18 months of ICM follow‐up.

## 2 METHODS

### 2.1 Participants

This prospective longitudinal study recruited 30 patients, aged 18–65 years, 25 (83.3%) males, with an unequivocal diagnosis HCM from the Region Gävleborg and University Hospital of Umeå in Sweden. The patients were identified from hospital databases using the diagnostic codes I42.1 and I42.2 and subsequently validated by the investigators using medical records. An estimated 5‐year risk of sudden cardiac death <6% according to the risk calculator was a prerequisite, since with higher risk an ICD should be considered, based on ESC guidelines.^9^ The exclusion criteria constituted of conditions known to cause hypertrophy: aortic stenosis (moderate to severe), metabolic disease (e.g., Anderson‐Fabry), syndromes (e.g., Noonan syndrome), and amyloidosis. A history of ischemic heart disease (myocardial infarction, percutaneous coronary intervention, coronary bypass grafting), electrophysiological intervention (pulmonary vein isolation, Maze surgery, VT ablation, ectopic atrial tachycardia ablation, or an implantable device (pacemaker, ICD, ICM) also excluded participation in the study. Patients with a history of an accessory pathway were excluded. Moreover, other exclusion criteria were: malignancy or other comorbidities with a life expectancy ≤5 years, renal clearance ≤40 ml/min (Cockcroft–Gault equation), systolic heart failure with ejection fraction ≤52%, pregnancy (or planned ≤18 months), drug addiction, severe psychiatric disease, and those not able to participate in 18 months follow‐up.

### 2.2 Implant and monitoring

All patients were implanted between August and December 2017. The implantation procedure was performed under local anesthesia (carbocaine with epinephrine) using the standard operation kit for Confirm Rx via a subcutaneous incision at the level of the fourth rib on the left side. The monitoring application, connected to home monitoring site Merlin, was installed on the patients' own or borrowed smartphone. The patient was instructed on how to use the application and report symptoms.

### 2.3 Definitions of arrhythmias

The ICM programming was standardized. VT detection was defined as 160 bpm during ≥8 intervals with high electrogram (EGM) priority, and the discriminator “sudden onset” was activated (onset delta 18%), and a bigeminy qualifier was programmed off. A potential AF episode was recorded based on ≥2 min duration (the shortest programmable duration). Bradycardia was recorded if the rate was ≤40 bpm or there was a pause ≥3.0 s. Patient‐activated recordings for symptoms had a high EGM priority and were set to record 6 min of pre‐trigger and 1 min of post‐trigger rhythms, with the first eight EGMs stored in memory. The maximal ventricular sensitivity was typically 0.15 mV but could be adjusted as necessary if R waves were low. The threshold start was programmed at 75%, sense refractory period 250 ms, and decay delay 60 ms.

### 2.4 Follow‐up

All participants completed the study with 18 months follow‐up as predefined. Technical support was sometimes requested when the patient wanted to change phones and reinstallation of the application was necessary. In a few patients, the study period had to be prolonged due to the temporary device disconnection. After the study period, all devices were explanted except in three cases, in which the clinician took over the continued monitoring.

### 2.5 Data analyses

All episodes were scrutinized by an expert review and categorized into atrial arrhythmia (atrial fibrillation, atrial flutter, ectopic atrial tachycardia), ventricular tachycardia (sustained VT and NSVT), sinoatrial block/arrest, and atrioventricular block (second or third degree). The differentiation between supraventricular arrhythmias and VT were based on general principles such as rate, regularity, sudden onset, and change in QRS morphology compared to a normal rhythm.

### 2.6 Statistical analyses

Data were described as numbers (*n*), percentages, ranges, percentiles, means, and standard deviations (SDs). To analyze the association between variables and outcomes, the *χ* ^2^ test and *t* test were employed, as appropriate. A two‐sided *p*‐value < .05 was considered significant, whereas associations with *p*‐values between .05 and .10 were considered a tendency. Kaplan–Meier estimates were used to describe the time from implant to the outcome, AF, and NSVT, respectively. For statistical analyses, SPSS version 25 (IBM) was used.

### 2.7 Ethics and registration

The study was approved by the Ethical Review Board in Umeå (document number 2017/13‐31) and registered at Clinical Trial Registration NCT03259113. Each patient was informed about the study in both oral and written form by a physician and included after written consent.

## 3 RESULTS

### 3.1 Patient characteristics

Before the study, the patients had been evaluated and followed‐up according to clinical routine, including echocardiography and Holter‐monitoring, typically every 1–2 years. The mean age of the 30 patients at the time of the ICM implant was 49.9 (*SD* 12.3) years. The vast majority were males (83.3%, *n* = 25) with a similar mean age (*p* = .796). Most patients had the maximum wall thickness localized in the septum (*n* = 26, 86.7%) and the remaining in the apex. Patient characteristics are summarized in Table 1. In each patient, the 5‐year estimated risk of sudden cardiac death was calculated; it ranged from 0.97% to 4.35% with a 25th, 50th, and 75th percentile 1.63%, 2.13%, and 3.00%, respectively. Only one patient had a risk score between 4% and 6%, that is, intermediate risk of SCD (sudden cardiac death) at baseline. The outcome on an individual level is summarized in Table 2.

**Table 1:** Baseline characteristics of 30 patients with hypertrophic cardiomyopathy

Age, mean (years)	49.9	*SD*, 12.9
Male	25	83.3%
Anthropometric data		
Weight (kg)	85.4	*SD*, 15.2
Length (cm)	177	*SD*, 10.1
Body‐mass index (kg/m^2^)	27.5	*SD*, 4.4
Five‐year risk of sudden cardiac death (%)	2.3	*SD*, 0.86
Risk factors		
Maximum wall thickness (mm)	19.3	*SD*, 5.6
Left atrial size (mm)	42	*SD*, 6.2
Max left ventricular outflow gradient	17.9	*SD*, 34.3
Family history of sudden cardiac death	4	13.3%
Non‐sustained ventricular tachycardia	2	6.7%
Unexplained syncope	2	6.7%
Cardiopulmonary exercise test		
Maximum work (W)	190	*SD*, 73.6
Maximum heart rate (bpm)	160	*SD*, 17.0
Maximum systolic blood pressure (mmHg)	187	*SD*, 33.7
Abnormal blood pressure response	0	0%
Ambulatory ECG		
Non‐sustained ventricular tachycardia	2	6.7%
Premature ventricular complex	14	46.7%
Atrial fib/flutter/ectopic ≥3 beats	0	0%
Premature atrial complex	11	36.7%
Genetic findings^a^		
MYBPC3	9	30%
MYH7	1	3.3%
TPM1	1	3.3%
Variant of unknown significance	2	6.7%
Laboratory findings		
NT‐proBNP (ng/L)	437	*SD*, 494
Hemoglobin (g/L)	148	*SD*, 10
S‐Potassium (mmol/L)	4.1	*SD*, 0.35
S‐Creatinine (mmol/L)	82	*SD*, 16
Troponin T > 15 (ng/L)	12	*SD*, 5.3
Myectomy	4	13.3%
Alcohol septal ablation	0	0%
Atrial fibrillation		
Paroxysmal	2	10.0%
Persistent	0	0%
Permanent	0	0%
Medication		
β‐blocker	19	63.3%
Calcium channel blocker	4	13.3%

^a^ In two patients no genetic analysis was performed.

**Table 2:** Individual patient 5‐year risk at implant and outcome during 18 months follow‐up using the insertable cardiac monitor Confirm Rx in hypertrophic cardiomyopathy

Sex, age at implant (year)	5‐year risk	Previous arrhythmia	Arrhythmia outcome	Time since implant (AF)	Time since implant (NSVT)
Male, 18	2.50		SA‐block		
Female, 27	4.35		SA‐block		
Male, 32	2.51		AF, SA‐block	3.6 months	
Male, 34	3.11		NSVT		4.3 months
Male, 37	2.21		NSVT		8.8 months
Male, 39	2.04		None		
Male, 41	1.70		None		
Female, 41	1.30		None		
Male, 41	2.05		SA‐block		
Male, 42	2.02		AF	1.0 months	
Male, 44	2.98		None		
Male, 46	1.90		AF, SA‐block	9.5 months	
Male, 46	2.43		None		
Female, 50	3.06	AF	AF	2.4 months	
Male, 54	2.37		SA‐block		
Male, 54	2.02		SA‐block		
Male, 54	1.64		SA‐block		
Male, 55	1.06		None		
Male, 55	3.77		AF	4.9 months	
Male, 57	1.38		SA‐block		
Male, 57	3.07		AF, NSVT, SA‐block	1.2 months	5.2 months
Male, 59	3.30	NSVT	NSVT, SA‐block		11.2 months
Male, 60	2.92		None		
Male, 60	1.48	AF	AF	1.3 months	
Female 61	0.99		None		
Female, 61	3.65		AF	3.8 months	
Male, 63	2.48	NSVT	NSVT		4.2 months
Male, 64	0.97		None		
Male, 65	1.67		NSVT, SA‐block		12.8 months
Male, 66	1.60		AF, NSVT, SA‐block	2.3 months	6.0 months

Abbreviations: AF, atrial fibrillation; NSVT, non‐sustained ventricular tachycardia; SA, sinoatrial.

### 3.2 Atrial fibrillation

During the 18‐month follow‐up, AF was detected in 30.0% (*n* = 9) of patients. In one of these patients, two episodes of atrial flutter were observed. The cumulative incidences of first device‐detected AF at 6 and 12 months were 26.7% and 30.0%, respectively, that is, the first episode was diagnosed within 1 year in all cases. In two of these patients, AF had been previously diagnosed before the implant. Thus, the device detected AF in 25% (*n* = 7) of patients without a history of AF. In one of the patients with a previously known AF, recurrent episodes occurred frequently and permanent AF developed despite treatment. In the remaining patients, the number of AF‐episodes ranged from 1 to 8 episodes with a total of 35 episodes, but they were all self‐terminating. Except for the patient who developed permanent AF, two patients reported symptomatic AF, of which one episode resulted in syncope (cycle length 352 ms, corresponding to 172 bpm). Patients with premature atrial complexes during 24‐hour ambulatory monitoring at baseline did not have significantly more AF than those without.

### 3.3 Non‐sustained ventricular tachycardia

At least one NSVT was diagnosed in 23.3% (*n* = 7) of the patients, all of the low‐risk patients with a 5‐year estimated risk score <4%. NSVT had been previously detected on Holter monitor in two patients, in whom NSVT was likewise detected in this study. Thus, the device detected NSVT in 5 of the 28 patients without a history of NSVT (17.9%). No episode of sustained VT (above 30 s) was reported. The cumulative incidences of first device‐detected NSVT at 6 and 12 months were 13.3% and 20.0%, respectively. Altogether, a total of 21 episodes occurred in all patients. Four patients had only 1 episode of NSVT (range 169–195 bpm; 16–23 beats); in two patients 2 episodes occurred, of which one was reported as symptomatic (presyncope); and in one patient a total of 11 episodes clustered during the same day followed by 2 episodes a few months later. Notably, in this latter patient, after the storm of NSVT, almost daily patient‐activated symptom recordings were transmitted, but with no correlation to any arrhythmia.

### 3.4 Sinoatrial block/arrest

**Figure 1:**
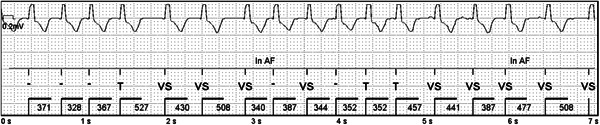
Atrial fibrillation detected by Confirm Rx

**Figure 2:**
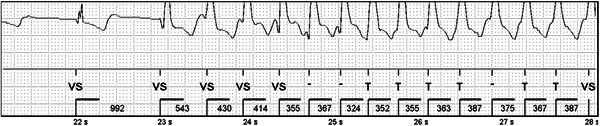
Non‐sustained ventricular tachycardia detected by Confirm Rx

In 43.3% (*n* = 13) of the patients, sinoatrial block/arrest or sinus bradycardia was observed at about 30–40 bpm. The longest pause was 3.1 s. Most episodes occurred during nighttime hours, and were asymptomatic. No atrioventricular block, second‐ (Mobitz type I/II) or third‐degree block was detected in any patient. No arrhythmias were detected in nine patients, and among the patients with arrhythmias there was some overlap. An example of arrhythmias are depicted in Figures 1 and 2.

## 4 DISCUSSION

### 4.1 Rationale

Among patients with HCM deemed at low risk according to the ESC risk calculator, a considerable arrhythmia burden was discovered by continuous long‐term monitoring. The ICM offers a complete, uninterrupted monitoring period in comparison to external loop recorders, where disconnections can occur, requiring follow‐up and technical support. The Confirm Rx, with its smartphone application for remote monitoring and symptom registration, proved to be feasible in this setting. The use of an ICM in HCM patients has been proposed in statements of research priorities^14^ to provide insights beyond routine evaluation in syncope and possibly recurrent palpitations.

### 4.2 AF is common

Thirty percent of the cohort was diagnosed with at least one episode of AF; a quarter of the cohort had newly diagnosed AF. The functional and structural abnormalities of the myocardium, especially low‐flow in the left atrial appendage, predispose patients to a prothrombotic condition, such that even AF of short duration implies stroke risk.^11^ By convention, an episode of at least 30 s is required for the AF diagnosis.^11^ An AF episode may be associated with symptoms or be asymptomatic. These latter episodes of asymptomatic or “silent AF” are common and constitute a similar risk for stroke.^11^ There are several technological approaches for the diagnosis, such as chest and thumb ECGs, skin patches, smartwatches, and blood pressure devices. While these novel devices show promise, the algorithms on which they rely are of variable quality. The role of such devices in HCM remains unknown, even though they are feasible, and direct costs are lower than with ICMs. The diagnostic yield depends on arrhythmia incidence, which may limit their use when arrhythmias are less frequent. The clinical management of device‐detected AF has been a matter of debate and may depend on the patient group and its risk factor profile as well as the duration and number of episodes.^15^ In a study of patients with pacemakers or ICDs, the presence of subclinical AF (>6 min) increased the risk of stroke by 2.5‐fold and conferred a fivefold increased risk of AF verified by 12‐lead ECG.^16^ In a systematic review of HCM patients, the prevalence of AF was 22.5%, and annual incidence 3.8%; the prevalence of stroke and annual incidence of thromboembolism were 27.1% and 3.8%, respectively.^2^ According to current guidelines, anticoagulation is indicated in HCM patients with a history of AF, even in the absence of conventional risk factors.^3^ Other more recent studies support this approach.^17^, ^18^ In our study, all patients with AF were recommended a non‐vitamin K antagonist oral anticoagulant.

### 4.3 NSVT may go unnoticed

A fifth of HCM patients who were considered low risk for sudden cardiac death turned out to have at least one episode of NSVT. Fortunately, no sustained ventricular arrhythmias occurred. In patients who likely represent more selected HCM patients with higher risk, similar prevalence has been observed on 24–48 h ambulatory ECG monitoring, and NSVT is considered to be a more pronounced risk factor at a younger age.^3^, ^6^, ^7^ It is unclear how the presence of NSVT on ICM should be interpreted in clinical practice. A thorough discussion of the findings and possibly individual reassessment using the risk calculator could be an approach. A dose increase of β‐blocker was recommended to patients with NSVT. After recalculation of the risk score with NSVT as a positive finding, no patient was above 6% risk. The clinicians were informed about the finding to judge whether an ICD should be advocated. To date, no patients were recommended an ICD, but these findings might be helpful in cases of borderline risk.

### 4.4 Bradycardia

Sinoatrial block/sinus arrest or sinus bradycardia were common, especially during nighttime, but did not meet the criteria for pacemaker implantation.^19^ These arrhythmias are likely due to increased vagal tone and does not necessarily imply pathological finding. Our study confirms the generally held belief that symptomatic bradycardia caused by sinus node dysfunction or atrioventricular block is uncommon in HCM. Nevertheless, when bradycardia was observed, it provided useful information for dose adjustment of pharmacological therapy, that is, β‐blockers and/or calcium‐channel antagonists.

### 4.5 Clinical perspectives

It is important to stress that diagnostic yield reflects patient selection. In an ICM study of 16 highly selected patients with advanced Anderson‐Fabry disease, significant arrhythmias were remarkably common.^20^ In HCM, current guidelines provide advice on management, and the potential role of the ICM in broader groups remains to be established. Nevertheless, device‐detected arrhythmias may have clinical consequences with regard to anticoagulation, risk stratification, initiation, and adjustment of pharmacological therapies, and they may help explain symptoms and provide valuable insight in terms of therapeutic decisions about imaging and patient counseling.

Future research of larger series of HCM patients would be welcomed, using ICMs allowing for analysis of subgroups or subsets, like patients who have undergone myectomy or alcohol septal ablation. Nowadays, an ICM can be easily implanted outside the electrophysiological laboratory and connected to a smartphone application for remote monitoring, which reduces cost compared to other monitoring systems.

### 4.6 Limitations

This is the first study on arrhythmia detection in HCM using an ICM, which allows the evaluation of the true incidence of arrhythmias. Still, an ICM offers single lead tracings, which may limit differentiation between supraventricular tachycardia and VT. ICM insertion is invasive and costly, so they are limited to selected patients in clinical practice. The findings in this study of unselected low‐risk patients need to be confirmed in larger cohorts and subgroups of HCM in different settings.

## 5 CONCLUSION

In unselected HCM patients deemed at low or intermediate risk for sudden cardiac death, 18 months evaluation using an ICM found that a considerable proportion of patients had arrhythmias: 30.0% AF, 23.3% NSVT, and 43.3% sinoatrial block/arrest or sinus bradycardia.

## AUTHOR CONTRIBUTIONS

**Peter Magnusson**: idea, design, recruitment, data collection, ECG‐analyses, writing, and project management. **Stellan Mörner**: design, recruitment, data collection, critical revision, and project management.

## ETHICS STATEMENT

The study was approved by Ethical Review Board in Umeå (document number 2017/13‐31) and registered at Clinical Trial Registration NCT03259113.
